# Molecular Modifications and Control of Processes to Facilitate the Synergistic Degradation of Polybrominated Diphenyl Ethers in Soil by Plants and Microorganisms Based on Queuing Scoring Method

**DOI:** 10.3390/molecules26133911

**Published:** 2021-06-26

**Authors:** Tong Wu, Yu Li, Hailin Xiao, Mingli Fu

**Affiliations:** 1 College of Environment, Energy of South China University of Technology, Guangzhou 510006, China; luxvnwolaopo@outlook.com (T.W.); esxiaohl@mail.scut.edu.cn (H.X.); 2 MOE Key Laboratory of Resources and Environmental Systems Optimization, North China Electric Power University, Beijing 102206, China

**Keywords:** polybrominated diphenyl ethers (PBDEs), molecular modification, synergistic degradation, molecular docking, molecular dynamics

## Abstract

In this paper, a combination of modification of the source and regulation of the process was used to control the degradation of PBDEs by plants and microorganisms. First, the key proteins that can degrade PBDEs in plants and microorganisms were searched in the PDB (Protein Data Bank), and a molecular docking method was used to characterize the binding ability of PBDEs to two key proteins. Next, the synergistic binding ability of PBDEs to the two key proteins was evaluated based on the queuing integral method. Based on this, three groups of three-dimensional quantitative structure-activity relationship (3D-QSAR) models of plant-microbial synergistic degradation were constructed. A total of 30 PBDE derivatives were designed using BDE-3 as the template molecule. Among them, the effect on the synergistic degradation of six PBDE derivatives, including BDE-3-4, was significantly improved (increased by more than 20%) and the environment-friendly and functional evaluation parameters were improved. Subsequently, studies on the synergistic degradation of PBDEs and their derivatives by plants and microorganisms, based on the molecular docking method, found that the addition of lipophilic groups by modification is beneficial to enhance the efficiency of synergistic degradation of PBDEs by plants and microorganisms. Further, while docking PBDEs, the number of amino acids was increased and the binding bond length was decreased compared to the template molecules, i.e., PBDE derivatives could be naturally degraded more efficiently. Finally, molecular dynamics simulation by the Taguchi orthogonal experiment and a full factorial experimental design were used to simulate the effects of various regulatory schemes on the synergistic degradation of PBDEs by plants and microorganisms. It was found that optimal regulation occurred when the appropriate amount of carbon dioxide was supplied to the plant and microbial systems. This paper aims to provide theoretical support for enhancing the synergistic degradation of PBDEs by plants and microorganisms in e-waste dismantling sites and their surrounding polluted areas, as well as, realize the research and development of green alternatives to PBDE flame retardants.

## 1. Introduction

Polybrominated diphenyl ethers (PBDEs) are widely used as brominated flame retardants (BFRs) in production and everyday life [1]. Due to long-term and large-scale use, many PBDEs directly or indirectly have spread through the air, water, soil, sediment, organisms and human bodies, posing a threat to the ecosystem and human health [2]. Especially in the underdeveloped areas such as Asia [2] and Africa [3]. The uncontrolled burning, disassembly and disposal of e-wastes in Nigeria cause a variety of environmental problems such as ground water contamination atmospheric pollution and water pollution [4]. Each year, large volumes of e-waste from Europe and North America are shipped to developing countries such as Ghana, Nigeria and South Africa [5,6]. In the 1980s, 70% of the world’s e-waste was disposed of in China, causing serious and persistent pollution by PBDEs [7]. The main areas polluted by PBDEs in China are the e-waste dismantling sites and their surrounding areas [8]. Additionally, the degradation of PBDEs mainly requires plants and microbes [9]. Therefore, it is of practical significance to study the synergistic degradation of PBDEs by plants and microorganisms (hereafter referred to as synergistic degradation) in soil. Chemometric methods and QSAR models based on computational chemistry are often used as key tools for pre synthesis and property prediction of novel compounds [10,11,12].

Plants play an important role in the natural degradation of PBDEs. Sun et al. [13] used pumpkin to degrade BDE-47 and found that BDE-47 was degraded into four polybrominated diphenyl ethers, including BDE-28, through metabolism in the plant tissues and rhizosphere. Huang et al. [14] selected six plants, such as maize, to treat BDE-209 polluted soil through pot experiments, and found that the removal rates of BDE-209 by the six plants ranged from about 12% to nearly 40%. Additionally, microorganisms also play an important role in the natural degradation of PBDEs. Tokarz et al. [15] found that the efficiency of anaerobic degradation of PBDEs increases with the number of bromine atoms, that is, the highly brominated PBDEs molecules are more unstable, more prone to degradation and easier to debromination reactions [16], i.e., the highly brominated PBDEs molecules. Kim et al. [17] discovered a strain of *Sphingomonas* in sediments, which could degrade BDE-3 and BDE-8 to produce bromophenol, catechol and other small molecules. (Structures of PBDEs that are mentioned in the introduction can be seen with Table S1 in Supplementary Materials).

The source modification based on molecular modification and the process control based on molecular dynamics are the focus and hotspot of the collaborative degradation of pollutants by plants and microorganisms. Gu et al. [18] explored the types of polychlorinated naphthalene (PCN) contaminated soil and determined the practicable scheme of combined remediation using an integrated method of genetic engineering and environmental remediation technology. Gu et al. [19] designed thirteen new proteins/enzymes, which significantly promoted the absorption, degradation and mineralization of the plant-microorganism combined remediation on PCN-contaminated soil. Moreover, the binding force of proteins/enzymes interacting with PCNs was the main index to evaluate the ability of plant-microorganism-combined remediation. Based on previous studies on the degradation of pollutants by microorganisms and plants, researchers have explored a new way to comprehensively evaluate the degradation of pollutants by microorganisms and plants.

To enhance the synergistic degradation of PBDEs in soil and explore the regulatory scheme that can effectively degrade PBDEs, in this study, a 3D-QSAR model for the synergistic degradation of PBDEs was established based on the queuing scoring method. Moreover, the template molecules were modified to obtain more suitable synergistic degradation and less polluting PBDE derivatives. Then, the similarities and differences in the degradation of the template molecules and the designed PBDE derivatives by plants and microorganisms were compared based on a molecular docking method. Finally, based on the Taguchi orthogonal experiment, a full factorial experimental design and molecular dynamics simulation, the effects of the degradation of template molecules by plants and microorganisms were simulated by adding different combinations of regulatory factors to the target soil. The aim was to determine the appropriate regulatory schemes for the synergistic degradation of PBDEs in soil for different combinations of various regulatory factors.

## 2. Materials and Methods

### 2.1. Determining the Binding Ability between PBDEs and Degrading Enzymes of Plants and Microorganisms—Molecular Docking

PBDEs in the soil can be degraded partially by nitrate reductase in *Zea mays* and ATP-binding cassette (ABC) protein in *Pseudomonas aeruginosa* [20,21]. A molecular docking method was implemented to determine the affinity between PBDEs and degrading enzymes in plants and microbes. The molecular structures were loaded into Discovery Studio 4.0 (BIOVIA, San Diego, CA, USA) package, while important functional enzyme receptors (PDB ID: 1CNF, 1L7V) of plants and microbes that degrade PBDEs, obtained from the Protein Data Bank (PDB), were selected as receptor molecules through the LibDock module. A function (stated as: “Find Sites from Receptor Cavities”) attached to a module (stated as: “Define and Edit Binding Site”) was used to search for the sites where PBDEs bind to degrading enzymes. Then the radius of the binding site was modified and defined. Finally, PBDEs were docked as ligands with the receptor protein by integrating the binding cavity formed by the receptor after obtaining the LibDock score, which determined the binding ability, i.e., the change in the degradation ability [22].

### 2.2. Construction of the Indices of Synergistic Degradation of PBDEs—Queuing Scoring Method

In recent years, the comprehensive evaluation of pollutants by comprehensive evaluation method is a hot research. According Averaging method, Threshold Method et al. (References on comprehensive evaluation of pollutant properties using mathematical methods can be seen with Table S2 in Supplementary Materials). In the Queuing scoring method, all evaluation units are queued up according to the merits and demerits of each evaluation index to obtain a sequence containing ‘*n*’ units and the score of each evaluation unit for every evaluation index (i.e., the individual score) is calculated separately [23].

While ranking the degradation indices of plants and microorganisms, if a molecule ranks ‘*k*’ among the docking score of all molecules (1 ≤ *k* ≤ *n*), its single item score (*SS*) for the degradation index is calculated as follows:

(1) SS=100−(k−1)/(n−1)×100=(n−k)/(n−1)×100

The first place is assigned a score of 100, while the last place is assigned a score of 0. The molecules in the middle are assigned scores between 100 and 0. Finally, the synergistic value (*CS*) of the docking scores between PBDEs and receptors containing plant (*PS*) and microbial (*MS*) degrading enzymes is obtained through the weighted arithmetic average of the single score for two degradation patterns, according to Formula (2).

(2) $$CS=(\sum_{j=1}^{m}SS_{j}\times W_{j})/\sum_{j=1}^{m}W_{j}$$

where $W_{j}$ is the docking score between each molecule and degrading enzyme of plants or microorganisms, and ‘*m*’ is the number of PBDEs involved in evaluating the synergistic degradation.

### 2.3. Molecular Modification to Facilitate the Synergistic Degradation of PBDEs in Soil—D-QSAR Model-Assisted Method

**Figure 1:**
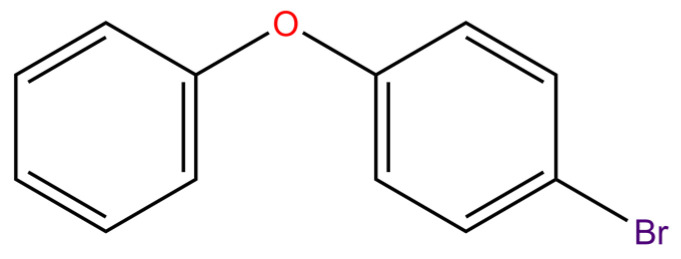
The structure of template molecule BDEs-3.

Among the homologs of PBDEs, the half-life of BDE-3 is the greatest [24], which means that the spontaneous degradation rate of BDE-3 is the lowest in its natural state. Thus, BDE-3 was selected as a template molecule for molecular modification (Figure 1). PBDEs were modified suitably to enhance the synergistic degradation by plants and microorganisms, i.e., to combine with degrading enzymes better. The aim was to obtain novel PBDE derivative molecules with higher synergistic degradation and less polluting to the environment. All the 3D-QSAR analyses were performed on the SYBYL-X 2.0 software (Tripos, Princeton, NJ, USA).

To achieve the above goals, a 3D-QSAR model was constructed with the synergistic value (*CS*) of the docking scores (for the docking of PBDEs to plant and microbial degrading enzymes) as the dependent variable determined by the queuing scoring method. The model simulated the synergistic degradation of PBDEs in soil, with the molecular structure of PBDEs used as the independent variable. A combination rule was followed during the construction of the model. The ratio of the number of molecules in the training set and test set was maintained at about 3:1 [25]. After the *CS* was imported into the software, the parameters of the 3D-QSAR model were automatically calculated by Sybyl-X 2.0 through the Autofill module. Partial least squares (PLS) analysis was applied to construct the relationship between the molecular structures of PBDEs and the *CS* [26]. Initially, the “Leave-One-Out” method was used to cross-validate the compounds in training set along with the cross-validation coefficient (*q*^2^), and the optimal principal component number (*n*) was calculated. Then, a “No Validation” regression analysis was performed to calculate the non-cross validation coefficient (*R*^2^), which was used for the internal validation of the 3D-QSAR model.

The external validation method (the most valuable validation method) was used to evaluate the predictive ability of the constructed model. By predicting the activity of independent test set compounds, the overall predictive ability of the 3D-QSAR model was externally verified [27,28]. The predictive ability of the model was expressed as $r_{pred}^{2}$, which was calculated by Formula (3).

(3) $$r_{pred}^{2}=1-\sum {\left(y_{i}-{\hat{y}}_{i}\right)}^{2}/\sum {\left(y_{i}-{\bar{y}}_{TR}\right)}^{2}$$

where $y_{i}$ represents the experimental value of molecules in the test set, ${\hat{y}}_{i}$ is the estimated value of molecules in the test set and ${\bar{y}}_{TR}$ is the average experimental value of molecules in the training set.

In this study, two single-factor 3D-QSAR models for the degradation of PBDEs by plants (PM) and microorganisms (MM) were constructed simultaneously to evaluate and validate the synergistic model (CM).

### 2.4. Regulatory Measures to Facilitate the SYNERGISTIC Degradation of PBDEs in Soil

#### 2.4.1. Preliminary Screening of Regulatory Factors That Facilitate the Synergistic Degradation of PBDEs in Soil—Taguchi Orthogonal Experimental Design

For the preliminary screening of experimental factors, the Taguchi orthogonal experimental design was used. This is a special orthogonal experimental method [29] that can arrange a large number of experimental factors in critical order. In order to simulate the effect of adding regulatory factors on the synergistic degradation of PBDEs by plants and microorganisms in soil, the L12 orthogonal test was used to design Taguchi test. The experimental design was carried out based on the natural degradation of PBDEs in the soil. A total of 11 factors (collectively referred to as regulatory factors) were used as the variables to generate the orthogonal experiment, and the addition of each variable was taken as the experimental level (1, 0). Of the 11 factors, six were common elements in the soil comprising carbon (carbon dioxide, glucose), nitrogen (ammonia-nitrogen, urea), oxygen (oxygen gas), phosphorus (phosphorus pentoxide, phosphate ester), magnesium (magnesium ion) and calcium (calcium ion) [30]; two were commonly used remediation agents for organically polluted soil, an oxidant (hydrogen peroxide) and a reducing agent (hydrogen sulfide) [31].

#### 2.4.2. Verification of Regulatory Schemes to Facilitate the Synergistic Degradation of PBDEs in Soil—Full Factorial Experimental Design

Based on the previous step, Taguchi orthogonal experiment screening, it is helpful to enhance the synergistic degradation of PBDEs by plants and microorganisms. The purpose of this article is to analyze the effect of adding different combinations of regulatory factors on the synergistic degradation of PBDEs by plants and microorganisms. The factorial experiment design can eliminate the high-order interaction between QNs molecules [22] and effectively screen the key factors that enhance the synergistic degradation of PBDEs by plants and microorganisms. The factorial experiment design uses the fixed effects model in Minitab DOE (Design of Experiment) software to analyze the contribution of each regulatory factor in the simulated added regulatory factor system.

#### 2.4.3. Verification of Regulatory Schemes to Facilitate the Synergistic Degradation of PBDEs in Soil—Molecular Dynamics

In this study, molecular dynamics simulation was performed on the Dell PowerEdge R7425 server using the GROMACS software. The number of composite indices of BDE-3 and its derivatives and the degrading enzymes was set as ‘1’. The energy minimization simulation was performed by the steepest gradient method. The pressure of the bath was set at 1 bar (at a constant standard atmospheric pressure) [25]. After assigning the synergistic degradation of PBDEs in the soil as the research target, the blank control group and the experimental group were set to simulate the binding of PBDE derivatives with degrading enzymes with the condition of adding regulatory factors. By simulating new combinations of regulatory factors, suitable combinations for the synergistic degradation of PBDEs in the soil were determined. No regulatory factors were added to the control group, while regulatory factors were added and combined to the experimental group. It was necessary to sample the equilibrium trajectory of the protein-ligand complex and calculate the binding free energy of the complex, protein and ligand, respectively, to calculate the binding free energy of MM/PBSA [25].

The binding free energy is calculated by the formula:

(4) $$G_{bind}=G_{complex}-G_{free-protein}-G_{free-ligand}$$

In solution, the binding free energy of the molecule can be calculated as:

(5) $$G=E_{gas}-TS_{gas}+G_{solvation}$$

where the solvation free energy can be decomposed into polar and non-polar free energy as:

(6) $$G_{solvation}=G_{polar}+G_{nonpolar}$$

## 3. Results and Discussion

### 3.1. Determination of Evaluation Indices for the Synergistic Degradation of PBDEs in the Soil Based on the Molecular Docking Method and the Queuing Scoring Method

Based on the molecular dynamics and queuing scoring method, synergistic evaluation indices for the molecular synergistic degradation of PBDEs were determined. The results of the evaluation indices are shown in Table 1.

In Table 1, PS represents the docking score of plant degradation, MS represents the docking score of microorganism degradation, CS represents the docking score of comprehensive degradation. Finally, CS group data is selected as the database of the 3D-QSAR model.

### 3.2. Molecular Modification and Evaluation Based on the 3D-QSAR Model to Facilitate the Synergistic Degradation of PBDEs in the Soil

#### 3.2.1. Construction of the 3D-QSAR Model to Facilitate the Synergistic Degradation of PBDEs in the Soil

**Figure 2:**
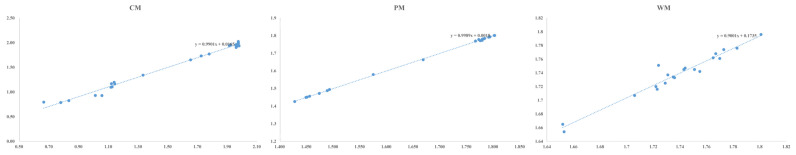
Scatter diagram of model training set and test set.

The results of the 3D-QSAR model evaluation are shown in Table 2. The results of the internal verification of the model showed that the best principal components (*n*) of the three CoMFA models were 4, 10 and 3, respectively. The cross-validation coefficients (*q*^2^) were 0.910, 0.904 and 0.882, respectively. The results indicated that the models had a good predictive capability [32]. The model is generally reliable when *q^2^* > 0.5 [33]. When (*R*^2^ − *q*^2^)/*R*^2^ is less than 25%, there is no over-fitting in the model [34]. To determine the external validation of the model, Equation (3) was used and the external predictive capability of the CoMFA models was evaluated. The results of the external validation based on Equation (3) showed that the interaction test coefficients *r*^2^_pred_ were 0.998, 0.999 and 0.998 (>0.6), respectively, indicating that the models had a good fit and predictive capability [35]. The scatter diagram of the model training set and test set is shown in Figure 2. (Horizontal comparison of model parameters can be seen with Table S3 in Supplementary Materials).

#### 3.2.2. Molecular Modifications to Facilitate the Synergistic Degradation of PBDEs in Soil

According to the three groups of models constructed (see Section 3.2.1), the contributions of the model force fields to the binding ability of PBDEs with the degradation enzymes were analyzed. The CM model contributed 29.70% to the three-dimensional fields and 70.30% to the electrostatic fields. The PM model contributed 44.00% to the three-dimensional fields and 56.00% to the electrostatic fields. Similarly, the contribution of the MM model was 40. 80% to the three-dimensional fields and 59.20% to the electrostatic fields. The spatial effect and the electrical distribution of the groups influenced the binding ability of the PBDE derivatives to the degradation enzymes; the electrical distribution was the most significant factor for binding. Figure 2 shows the three-dimensional contour maps of the three groups of the CoMFA models with the target molecule BDE-3 as a reference. The block diagrams in different colors show the effects of the three-dimensional field and the electrostatic field on the natural degradation capacity of BDE-3 [36].

**Figure 3:**
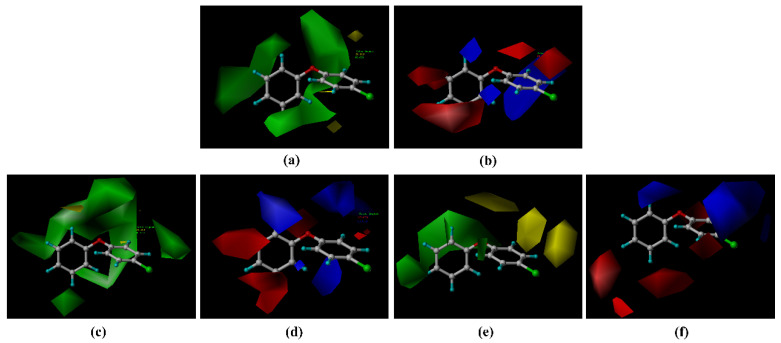
Contour maps of the model contour maps. CM: (**a**,**b**); PM: (**c**,**d**); MM: (**e**,**f**).

As shown in Figure 3, in the three-dimensional field, the green area indicates that the introduction of a large group can enhance the natural degradation capacity of the pollutant, while the yellow area indicates that the introduction of a large group can diminish the natural degradation capacity of the pollutant. In the electrostatic field, the blue area indicates that the addition of positively charged groups can enhance the natural degradation capacity and the red area indicates that the addition of negatively charged groups can enhance the natural degradation capacity [36]. Based on the above analysis of the three-dimensional contour maps, a total of 30 PBDE derivatives were designed by selecting the modified groups that match the corresponding properties, as shown in Table 3. Molecular information of PBDEs derivatives as shown with Table S4 in Supplementary Materials.

In the synergetic degradation model (CM), the predicted values of 13 PBDE derivatives, such as BDE-3-1, BDE-3-2 and BDE-3-3, were elevated (1.16-43.88%) compared to the target molecule and BDE-3-10 showed the greatest increase (43.88%) (Table 3). In the plant degradation model (PM), the predicted values of six PBDE derivatives, such as BDE-3-1, BDE-3-7 and BDE-3-11, improved significantly (2.09-25.19%) compared to the target molecule, and BDE-3-19 showed the greatest increase (25.19%). In the microbial degradation model (MM), the predicted values of 14 PBDE derivatives such as BDE-3-1, BDE-3-3 and BDE-3-4 improved significantly (8.14-37.09%) compared to the target molecule, and BDE-3-11 showed the greatest increase (37.09%). In summary, from the predictions of the three models, all 11 PBDE derivatives, such as BDE-3-1, BDE-3-2 and BDE-3-4, showed greater synergistic degradation and the degrading ability of the plant and microbial single factors were enhanced. Therefore, 11 PBDE derivatives (BDE-3-1, BDE-3-2, BDE-3-4, BDE-3-5, BDE-3-6, BDE-3-7, BDE-3-8, BDE-3-9, BDE-3-10, BDE-3-13, BDE-3-19) were selected for the next step, the evaluation of the properties of PBDE derivatives that may affect the environment.

#### 3.2.3. Evaluation of the Functionality and the Environmental Impact of PBDE Derivatives to Facilitate the Synergistic Degradation of PBDEs in Soil

Flame retardants weaken or stop combustion by preventing the chain branching reaction [28]. Studies have shown that PBDEs decompose hydrogen bromide (HBr) during combustion, and the highly reactive H-and OH-radicals generated during the combustion of polymeric materials become trapped and react with them, eventually leading to slowing or stopping of the combustion [37]; the core of the reaction is the dissociation of the C-Br bond. Therefore, the efficiency of halogen-based flame retardants is related to the strength of the C-Br bond, and the efficiency of bromine-based flame retardants with low C-Br bond energy is often higher than that of chlorine-based flame retardants. Bromine-based flame retardants can produce bromine radicals and hydrogen bromide that can minimize the flame. Therefore, the C-Br bond dissociation enthalpy (*R-Br*) is selected as the parameter to evaluate the efficiency of the flame retardant in this study. Taking the *R-Br*→*R+Br* reaction as an example, the specific equations for calculating the bond dissociation enthalpy are as follows [38,39].

(7) $$R-Br=H_{298}^{0}(R)+H_{298}^{0}(Br)-H_{298}^{0}(RBr)$$

(8) $$H_{298}^{0}=E+\Delta ZPE+\Delta H_{trans}+\Delta H_{rot}+\Delta H_{vib}+RT$$

where ΔZPE is the zero-point energy, $\Delta H_{trans}$, $\Delta H_{rot}$ and $\Delta H_{vib}$ are the energy contributions by translation, rotation and vibration, respectively; *T* is the specific temperature (K). For the atoms and free radicals involved in the reaction, the B3LYP/6-31G (d, p) calculated energy level was used to optimize without imaginary frequencies. In addition, B3LYP/6-311G (d, p) calculated energy level was selected to calculate their single point energies, which were previously shown to be accurate in calculating carbon-halogen bond dissociation energies [40]. In this paper, the flame retarding parameters of PBDE derivatives were calculated on the Gaussian 09W software package and the Gaussview 5.0 program. Then, the EPI database prediction method was used to predict the biological toxicity (EC50) of PBDE derivatives, the molecular enrichment (log BCFs) of the derivatives and the long-distance migration (VP) of the derivatives [41], to determine the PBDE derivatives with good flame retardancy and lower environmental pollution. The results are shown in Table 4.

All 11 BDE-3 derivatives showed better flame retardancy than the target molecule (Table 5). Among them, BDE-3-10 had the highest predicted flame retardancy value (96.357). Even though the overall improvement in flame retardancy was not significant, all 11 BDE-3 derivative molecules maintained a positive trend in flame retardancy. For biotoxicity, the predicted toxicity of six PBDE derivatives, including BDE-3-4, BDE-3-5 and BDE-3-6, was lower than the target molecule (28.29–94.12%). Among them, the predicted value of toxicity of the BDE-3-7 derivative was the lowest (0.047), and the toxicity values of other derivatives were higher than the target molecule. For bioconcentration, the predicted values of all the derivatives were lower (29.10–91.54%) than the values of the target molecules. For long-distance migration, all PBDE derivatives had lower values than the predicted long-distance migration of the target molecule (90.18–100.00%). In summary, among the PBDE derivatives designed by the CoMFA models to facilitate the synergistic degradation of PBDEs in soil, five derivative molecules, BDE-3-4, BDE-3-5, BDE-3-7, BDE-3-13 and BDE-3-19, not only had enhanced synergistic degradation but also were less polluting to the environment. Thus, they can be recommended as substitutes for PBDEs.

According to the prediction of this paper and various property models, the newly designed derivatives have passed the evaluation of environmental friendliness and functionality, and can also be environmentally friendly on the premise of ensuring the function. The toxicity metabolism model prediction of derivative molecules was supplemented (Table S5-1,2,3). As shown in Table S5-1,2,3, compared with LEV, LEV derivatives designed and modified based on 3D-QSAR showed the same or weaker toxicity evaluation, and few individual evaluation items of a few derivative molecules showed a little upward. Therefore, we believe that the LEV derivatives designed and modified based on 3D-QSAR are feasible in application (The Table S5-1,2,3 can be seen in Supplementary Materials).

### 3.3. Screening of Regulatory Factors and Regulatory Schemes to Facilitate the Synergistic Degradation of PBDEs in Soil

As an important part of the environment and the ecosystem, the soil is rich in elements and nutrients needed by plants and microorganisms [30], but it is also vulnerable to pollution, including organic pollution by PBDEs [7]. PBDEs are mainly degraded by plants and microbes [9]. Additionally, adding oxidants and reductants to the contaminated plots for the chemical removal of organic pollutants from the soil is one of the conventional methods for remediation of soil organic pollution [31]. In this paper, nine substances of six types of elements in the soil were selected as regulatory factors for simulation-based analysis. These were carbon (carbon dioxide, glucose), nitrogen (ammonia-nitrogen, urea), oxygen (oxygen), phosphorus (phosphorus pentoxide, phosphate ester), magnesium (magnesium ion) and calcium (calcium ion) [30]. Additionally, two common remediation agents for soil organic pollution, an oxidant (hydrogen peroxide) and a reductant (hydrogen sulfide) [31] were also used.

#### 3.3.1. The Preliminary Screening of Regulatory Factors to Facilitate the Synergistic Degradation of PBDEs in Soil Based on Taguchi Orthogonal Experiment and Molecular Dynamics Simulation

In this study, nine substances of six types of elements in the soil were selected as factors for the Taguchi orthogonal experiment. The compounds were assigned alphabetical identities as follows: carbon dioxide: A, glucose: B, ammonia-nitrogen: C, urea: D, oxygen: E, phosphorus pentoxide: F, phosphate ester: G, magnesium ion: H, calcium ion: I, hydrogen peroxide: J and hydrogen sulfide: K. A molecular dynamics simulation assisted by Taguchi orthogonal experiment was performed with two levels (‘0’ represented no addition and ‘1’ represented addition), and the average of Signal to noise ratio (SNR) and SNR range of the results were verified as evaluation criteria (Table 5).

The analysis showed that, for plants, the regulatory factors (in descending order of importance) that enhanced the degradation capacity of PBDEs were magnesium ion, phosphorus pentoxide, carbon dioxide, phosphate ester, ammonia-nitrogen, urea, hydrogen peroxide, calcium ion, oxygen, hydrogen sulfide and glucose. Among them, magnesium ion, phosphorus pentoxide, carbon dioxide, phosphate ester, ammonia-nitrogen and urea had a relatively greater impact on the SNR of the plant groups. Therefore, they were classified as the divergence factors for plant groups. The analysis indicated that the metal ions in the soil and the appropriate N/P ratio can promote the degradation of PBDEs by plants. When plants are supplied with a moderate amount of carbon dioxide, photosynthesis is enhanced. This allows plants to convert inorganic substances to organic matter that is suitable for cell and protein synthesis and, thus, accelerates the transport and degradation of pollutants in the soil [43]. Studies have shown that nitrogen and magnesium are essential for the biosynthesis of plant molecules such as chlorophyll [43]. Potassium and phosphorus are involved in carbohydrate metabolism, and their deficiency can affect the transformation and transportation of carbohydrates, indirectly affecting photosynthesis. Additionally, phosphorus also participates in the transformation of intermediates and energy transfer during photosynthesis, significantly affecting the process. Photosynthesis promotes the growth of plants, which in turn promotes the processes of absorption, transformation and degradation of related substances in the soil.

For microorganisms, the regulatory factors (in descending order of importance) in the system that enhanced microbial degradation of PBDEs were urea, glucose, carbon dioxide, phosphate ester, phosphorus pentoxide, magnesium ion, oxygen, ammonia-nitrogen, hydrogen peroxide, calcium ion and hydrogen sulfide. Among them, urea, glucose, carbon dioxide, phosphate ester, phosphorus pentoxide and magnesium ions had a relatively large impact on the SNR, so they were classified as the divergence factors of microorganisms. Appropriate N/P ratio and metal ions in the soil affect the survival of microorganisms, and external carbon sources can effectively improve the degradation rate of residual pollutants by soil microorganisms, which is consistent with the conclusions of Chen et al. [20]. Cheng et al. [44] showed that, compared to aerobic conditions, degradation of PBDEs in the soil occurs more by anaerobic microorganisms under anaerobic conditions. Therefore, the conversion of the microbial community in the soil from aerobic to anaerobic is suitable for the degradation of PBDEs by anaerobic microorganisms in the soil.

Since carbon dioxide, urea, phosphorus pentoxide and phosphate ester are divergence factors common to both plants and microorganisms, these factors may have relatively profound effects on enhancing the synergistic degradation of PBDEs and could be further used as factors for a full factorial experimental design.

#### 3.3.2. Screening of Regulatory Schemes to Facilitate the Synergistic Degradation of PBDEs in Soil Based on Molecular Dynamics Simulation

Four regulatory factors (carbon dioxide: A, urea: D, phosphorus pentoxide: F and phosphate ester: G) that were selected by the Taguchi orthogonal experiment and facilitated the synergistic degradation of PBDEs in soil were used as factors for the analysis of the regulatory scheme. A full factorial experimental design with four factors and two levels (‘0’ represented no addition and ‘1’ represented addition) was performed to generate a total of 32 groups, which included blank control groups of regulatory combinations that facilitated the synergistic degradation of PBDEs in the soil (Table 6). For the analysis, the BDES-3-19 derivative molecule was taken as an example, and the dynamic combinations of the BDES-3-19 derivative molecule with 1CNF and 1L7V were simulated under the conditions of adding different combinations of regulatory factors (Table 6) to determine the degree of the effect of the regulatory schemes on the synergetic degradation of PBDEs in the soil.

The molecular dynamics simulation showed that the binding energy of the blank control combination in the plant and microorganism degradation groups were −39.964 kJ/mol and −10.186 kJ/mol, respectively. In the plant simulation experimental groups, except for the dynamic simulation effect of the experimental group No. 1, 2, 3 and 14, the binding ability of the remaining 11 groups decreased (−59.97–132.53%) compared to the blank control group. Among them, the effect of the experimental group No. 13 was the most significant, with a binding energy of −92.927 kJ/mol (increased by 132.53%). In the microbial simulation experimental group, except for the group No. 2, 4, 5, 7, 9, 11 and 12, the binding ability of the remaining 8 groups decreased compared to the blank control group (−50.82–298.90%). Among them, the effect of experimental combination No. 15 was the most significant, with a binding energy of −40.632 kJ/mol (increased by 298.90%). The synergistic analysis showed that the regulatory schemes could enhance the synergistic degradation of PBDEs in the soil, i.e., the binding energy of both plant and microorganism groups increased compared to the control group No. 6, 10, 13 and 15. Based on the four regulatory schemes mentioned above, the average increase in the rates of synergistic degradation of PBDEs in soil were 118.47%, 92.11%, 117.33% and 200.90%, indicating that regulatory scheme No. 15 was the best for the synergistic degradation of PBDEs in the soil.

For plants, a moderate increase in carbon dioxide promotes photosynthesis, the development of underground roots and the synthesis and secretion of root exudates. Chekol et al. [45] showed that the development of plant roots and root exudates enhances the degradation of contaminants in the soil. Since the 1950s, carbon dioxide fertilization of plant roots and the surrounding soil environment had been one of the conventional means to promote crop growth [46]. For microorganisms, carbon dioxide is transported to the surface of the soil through carbon dioxide fertilization, and an anaerobic environment is gradually formed in the soil, which allows anaerobic microorganisms to predominate. This accelerates the degradation of PBDEs by anaerobic microorganisms in the soil. Thus, artificially increased carbon dioxide in the vicinity of plants might increase the synergistic degradation of PBDEs in the soil under appropriate conditions.

### 3.4. Horizontal Comparative Analysis of the Mechanism of Degradation by Plants and Microorganisms before and after Molecular Modification of PBDEs Based on Molecular Docking Technology and Molecular Dynamics

**Figure 4:**
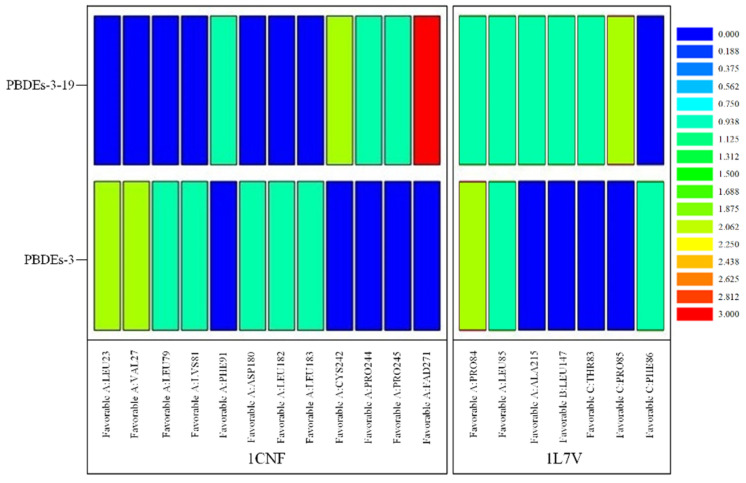
Heat map of significant amino acids in degradation of PBDEs by plants and microorganisms based on molecular docking technology.

Based on the docking results of BDE-3 and PBDE derivatives (taking BDE-3-19 as an example) with two degradation enzymes, the horizontal mechanism of degradation of PBDEs by plants and microorganisms before and after modifications was analyzed. Figure 4 shows the amino acids, which aid the enzymes to bind to the PBDEs.

**Figure 5:**
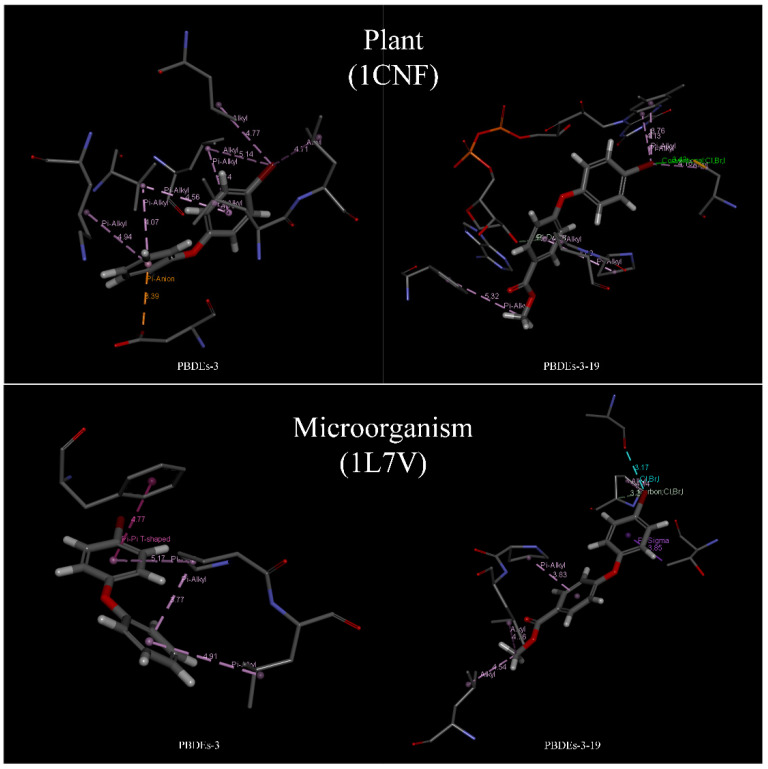
Degradation mechanism of PBDEs and its derivatives by plant and microorganism based on molecular docking technology.

The information of the bond type and bond length for the docking of BDE-3 molecule and BDE-3-19 derivative molecule with two kinds of degrading enzymes was visualized (Figure 5), and the relevant data in Figure 4 were statistically analyzed, as shown in Table 7.

From the point of view of the bond length between molecules and proteins, for the degradation of PBDEs by plants, the binding of PBDEs to 1CNF protein involved 12 kinds of amino acids, such as LEU23, VAL27 and LEU79 (see Figure 3). Remarkably, seven kinds of amino acids, such as LEU23, VAL27 and LEU79, were involved in the binding of BDE-3 to 1CNF protein, and the number of important amino acids was nine. Seven amino acids, such as LEU23, VAL27 and LEU79, were involved in the binding of BDE-3 to 1CNF protein, and the number of important amino acids was eight. According to the analysis (Figure 4 and Table 7), the average bond length of BDE-3-19 derivatives bound to the important amino acids decreased from 4.61 to 4.19, with a decrease of 9.10%. The shorter the bond length, the stronger the ability to degrade [32]. For the degradation of PBDEs by plants, the average bond length of BDE-3-19 derivatives combined with 1CNF protein was lower than that of BDE-3, indicating that the BDE-3-19 derivatives were more degradable than those of BDE-3. In microbial degradation, seven amino acids, such as PRO84, LEU85 and ALA215, were involved in the binding of PBDEs to 1L7V protein (Figure 3). Among them, three amino acids, such as PRO84 and LEU85, were involved in the binding of BDE-3 with 1L7V protein, and the number of important amino acids was four. Six amino acids, including PRO84, LEU85 and ALA215, were involved in the binding of BDE-3 with 1L7V protein, and the number of important amino acids was seven. Additionally, the average length of the interaction bond with the important amino acids decreased from 4.66 to 3.93 when BDE-3 and BDEs-3-19 derivatives were bound to 1L7V protein, with a decrease in bond length by 15.42% (Figure 4 and Table 7). For the microbial degradation of PBDEs, the average bond length of BDE-3-19 derivatives bound to 1L7V protein was lower than that of BDE-3, indicating that BDE-3-19 derivatives had stronger degradation by microorganisms than BDE-3.

Regarding the bond type for the interaction between molecules and proteins, BDE-3-19 derivatives produced halogen bonds (Br bond) when combined with plant and microbial degradation enzymes, but BDE-3 molecules could not. This is a significant difference between BDE-3 and BDE-3-19, which indicates that BDE-3-19 derivatives designed using the CoMFA model can stimulate the binding of a Br-group with the degrading enzyme of plants and microbes. This promotes the formation of higher binding energy, i.e., the degradation effect is stronger for BDE-3-19 derivatives than for BDE-3.

The molecular structure before and after the modification was analyzed. Zhang et al. [47] found that higher brominated PBDEs with greater lipophilicity have better transport and degradation properties than their lower halogenated homologs, i.e., the molecular lipophilicity is positively correlated with the effects of transportation and degradation. The higher brominated PBDE homologs have stronger lipophilicity than the lower halogenated ones, which is consistent with the molecular scheme of PBDE derivatives modified by the three-dimensional equipotential diagram; methyl formate is introduced into the modified site of BDE-3 to form BDE-3-19 derivatives with stronger lipophilicity. Considering the properties of amino acids, the ratio of hydrophobic amino acids before and after modification was 3:3 in plants and 4:6 in microorganisms. According to the analysis (Table 7), although the proportion of hydrophobic amino acids involved in the binding was the same, the average bond length was shortened, i.e., the binding ability was enhanced. During microbial degradation, more hydrophobic amino acids were involved in the binding process when the degradation enzyme combined with the PBDE derivatives, and the average bond length was shortened, which implies that the ability to degrade the PBDE derivatives was enhanced.

**Figure 6:**
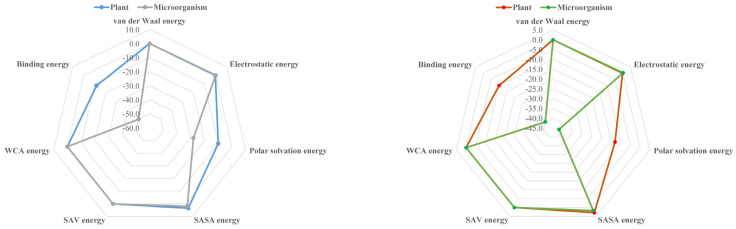
Comparison of binding energy and different force energy of PBDEs with plant and microbial degradation proteins before and after modification.

It can be seen from Figure 6 that the absolute value of binding energy between plant and microbial degradation protein and pbdes-3-19 molecule is significantly higher than that of template molecule, indicating that pbdes-3-19 derivative molecule is more easily degraded by plant and microorganism than template molecule. The binding energy is the sum of van der Waals energy, electrostatic interaction energy, polar solvation energy, Sasa energy, sav energy and WCA energy. The change rates of van der Waals energy, electrostatic interaction energy, polar solvation energy, Sasa energy, sav energy and WCA energy are 0.00%, −18.25%, −23.71%, −16.58%, 0.00% and 0.00%, respectively, when pbdes-3-19 derivative interacts with degradation protein. The change rate of polar solvation energy is the largest, which indicates that polar solvation energy is the main reason for enhancing the interaction between plant and microbial degradation proteins and pbdes-3-19 derivatives, that is, reducing the polar solvation energy properly in the process of plant and rhizosphere microbial degradation of PBDEs can improve its degradation ability.

## 4. Conclusions

In this study, the queuing scoring method was used to ingrate the 3D-QSAR model, molecular docking, molecular dynamics simulation. A full factorial experiment was conducted to determine the PBDE derivatives suitable for the synergistic degradation by plants and microorganisms in the soil. The derivatives were modified to minimize pollution at the source, and the regulatory schemes that could effectively enhance the degradation of the derivatives by plants and microorganisms were determined for process control. The molecular substitutes of PBDE flame retardants demonstrated in this study provide theoretical support for the replacement of flame retardants by easily degradable and environmentally friendly alternatives-in other words-it can bring new exploration for PBDEs pollution in soil environment of electronic waste disposal site from the perspective of source modification and process control. In future research, we should also pay attention to the effect of trace elements including iron in soil on the synergistic degradation of PBDEs by plants and microorganisms, and we should also analyze the synergistic degradation of PBDEs by specific plants and their root microflora in the future.

## Figures and Tables

**Table 1:** Synergistic evaluation indexes for the synergistic degradation of PBDEs ^a^.

No. ^b^	PS	MS	CS	No.	PS	MS	CS	No.	PS	MS	CS	No.	PS	MS	CS
1	50.733	55.710	62.419	54	44.215	38.509	14.237	102	49.677	56.691	63.460	154	28.227	58.385	55.058
2	57.837	54.414	70.497	55	59.243	63.288	93.809	103	48.879	59.590	68.261	155	45.577	49.031	30.561
3	54.702	53.951	74.645	56	52.388	52.759	56.244	104	51.399	43.820	39.281	158	33.236	55.042	40.348
4	53.765	58.411	76.761	57	46.600	56.159	53.336	105	22.051	66.738	75.164	160	49.039	47.847	34.447
5	51.594	54.430	59.291	58	52.568	66.686	86.330	106	37.133	55.909	44.280	161	43.484	53.569	37.484
6	54.788	58.776	79.569	59	60.472	60.682	93.996	107	40.979	58.222	52.755	162	43.448	53.694	37.567
9	56.667	58.159	80.267	60	62.142	60.081	93.801	108	50.356	52.020	48.741	163	52.614	55.499	66.179
10	48.301	48.648	34.540	61	60.028	55.127	78.122	109	57.796	60.055	86.599	164	43.981	43.946	17.431
11	56.974	58.361	81.129	62	47.218	45.482	26.500	110	50.426	48.015	39.848	166	44.549	48.599	26.236
12	59.214	59.652	88.565	63	53.948	60.093	81.574	111	60.748	54.667	78.467	167	36.432	59.307	56.086
13	57.526	62.455	90.223	64	55.251	57.275	75.959	112	42.758	50.425	27.825	168	45.575	48.689	28.220
14	46.754	53.730	43.984	65	50.753	49.440	46.755	113	41.305	50.123	25.711	170	44.321	49.409	29.298
15	60.261	48.748	65.275	66	62.080	60.168	94.067	114	45.632	52.818	39.093	171	44.514	53.524	39.336
16	55.174	53.059	62.587	67	55.945	63.837	89.974	115	44.264	50.525	31.501	172	28.530	54.541	37.749
18	51.739	61.670	81.201	68	49.737	55.682	59.719	116	60.087	47.239	61.154	173	39.364	48.431	18.817
19	50.613	53.869	55.063	69	43.235	51.439	29.886	117	57.489	54.592	70.919	174	46.630	43.739	22.688
20	61.828	56.043	83.315	70	53.591	57.129	72.018	118	46.867	55.849	52.467	175	29.743	47.470	13.265
21	59.743	47.605	60.642	71	48.967	55.600	55.668	119	41.126	51.845	29.131	176	52.210	47.876	45.561
22	56.985	61.494	88.196	72	34.005	61.398	62.223	120	23.833	32.945	0.240	177	52.552	55.468	65.328
23	46.574	51.513	36.415	73	48.682	37.079	25.795	121	37.530	39.004	6.780	179	48.790	44.680	29.598
24	50.741	48.690	43.771	74	47.394	53.408	44.307	122	50.160	57.683	66.126	180	43.234	51.712	30.582
25	57.937	61.921	90.695	75	42.093	56.075	46.193	123	49.449	57.237	62.548	181	43.164	56.096	47.201
26	55.000	66.484	88.768	76	57.124	51.931	63.293	124	49.217	52.803	46.521	182	47.599	50.822	38.525
27	48.062	54.728	50.537	78	57.964	58.741	85.128	125	35.000	45.774	13.029	184	47.169	50.452	36.552
29	55.790	53.374	64.633	79	52.080	58.986	75.962	126	59.742	54.379	73.484	185	34.935	46.142	13.124
31	55.890	52.077	62.105	80	55.455	59.350	82.678	128	44.885	49.017	29.168	186	46.193	42.368	21.388
33	57.809	54.456	70.747	81	63.311	60.652	94.955	131	37.731	52.188	30.241	187	28.077	46.347	11.322
34	54.998	62.107	86.001	82	40.683	55.810	43.790	132	48.683	54.837	52.710	188	42.491	45.731	16.529
35	59.593	57.332	83.023	83	53.855	54.972	66.221	133	52.406	57.826	73.068	189	43.676	53.130	36.085
36	52.978	56.362	70.563	84	47.792	46.413	29.600	136	55.272	43.873	48.766	190	45.756	52.131	37.782
37	63.410	58.137	89.614	86	47.246	54.512	47.469	138	37.551	55.567	42.118	191	42.498	53.366	34.827
38	55.095	58.726	79.796	87	44.090	63.495	67.402	139	34.033	56.214	46.441	192	49.392	39.556	29.639
39	58.382	64.565	93.673	88	31.567	56.544	47.519	140	49.381	51.854	44.752	193	40.695	44.963	13.686
40	53.988	54.565	65.097	89	50.567	45.006	36.692	141	54.419	48.726	52.452	195	48.284	48.900	35.669
41	59.806	48.917	64.348	90	51.033	57.709	70.135	142	49.549	47.546	36.330	196	51.013	49.047	46.589
42	50.645	53.764	55.049	91	58.794	52.697	67.806	143	44.001	39.951	15.026	197	31.670	47.313	13.816
43	58.173	63.262	92.168	92	51.110	57.428	69.475	144	30.790	59.308	57.608	198	30.236	41.261	5.979
44	60.472	60.682	93.996	93	38.732	50.418	25.175	145	44.858	45.424	21.100	200	45.785	40.797	20.186
45	50.950	48.487	43.310	94	49.677	56.691	63.460	146	47.708	48.258	31.664	201	45.450	38.554	17.126
48	59.166	61.223	90.877	95	51.612	39.231	38.738	147	51.697	54.749	62.187	202	47.098	34.293	21.403
49	50.075	59.706	72.572	96	50.716	44.696	37.174	148	43.867	43.268	16.047	203	31.091	48.457	16.630
50	49.330	55.678	56.752	97	51.033	57.709	70.135	149	26.750	40.848	4.596	207	45.193	49.078	30.333
51	49.461	52.917	48.494	98	49.576	51.478	45.294	150	49.542	50.205	42.800				
52	60.068	41.767	58.896	99	48.567	58.439	64.597	151	42.035	39.152	10.233				
53	48.198	57.865	61.615	101	54.120	49.688	54.593	152	47.398	40.260	23.944				

^a^: The single-factor data were obtained from the scoring values of the results of PBDEs dock with plant and soil degradation enzymes based on molecular docking method. ^b^: Molecules with missing serial numbers are PBDEs that have not successfully docked with one or all of the plant or microbial degradation enzymes. So, it will not be discussed here.

**Table 2:** The evaluation parameters of single factor CoMFA models to facilitate the synergistic degradation of PBDEs in soil.

Model	*q* ^2^	*n*	*SEE*	*R* ^2^	*F*	*Q* ^2^	*CSDEP*	*dq*^2^/*r*^2^*yy*	*r* ^2^_pred_
CM	0.910	4	0.063	0.988	312.127	0.732	0.053	1.039	0.998
PM	0.904	10	0.001	1.000	30,581.376	0.356	0.148	1.051	0.999
MM	0.882	3	0.016	0.984	210.388	0.870	0.060	1.025	0.998

**Table 3:** Prediction of degradation capacity of PBDEs derivatives.

Compounds	CM		PM		MM	
	Pred.	Relative Error (%)	Pred.	Relative Error (%)	Pred.	Relative Error (%)
BDEs-3	74.645	-	54.702	-	53.951	-
BDEs-3-1	90.157	20.78	60.395	10.41	59.704	10.66
BDEs-3-2	75.509	1.16	61.094	11.69	68.707	27.35
BDEs-3-3	95.499	27.94	51.523	−5.81	70.958	31.52
BDEs-3-4	96.828	29.72	57.677	5.44	63.680	18.03
BDEs-3-5	95.060	27.35	57.280	4.71	62.087	15.08
BDEs-3-6	77.446	3.75	55.847	2.09	61.235	13.50
BDEs-3-7	104.713	40.28	63.826	16.68	72.277	33.97
BDEs-3-8	106.414	42.56	58.210	6.41	63.680	18.03
BDEs-3-9	81.846	9.65	59.429	8.64	72.444	34.28
BDEs-3-10	107.399	43.88	57.810	5.68	62.087	15.08
BDEs-3-11	65.464	−12.30	65.013	18.85	73.961	37.09
BDEs-3-12	32.885	−55.94	61.518	12.46	70.469	30.62
BDEs-3-13	100.231	34.28	60.395	10.41	63.096	16.95
BDEs-3-14	30.409	−59.26	45.082	−17.59	51.880	−3.84
BDEs-3-15	30.409	−59.26	45.082	−17.59	51.880	−3.84
BDEs-3-16	30.409	−59.26	45.082	−17.59	51.880	−3.84
BDEs-3-17	30.409	−59.26	45.082	−17.59	51.880	−3.84
BDEs-3-18	30.409	−59.26	45.082	−17.59	51.880	−3.84
BDEs-3-19	90.910	21.79	68.479	25.19	68.345	26.68
BDEs-3-20	30.409	−59.26	45.082	−17.59	51.880	−3.84
BDEs-3-21	30.409	−59.26	45.082	−17.59	51.880	−3.84
BDEs-3-22	30.409	−59.26	45.082	−17.59	51.880	−3.84
BDEs-3-23	30.409	−59.26	45.082	−17.59	51.880	−3.84
BDEs-3-24	30.409	−59.26	45.082	−17.59	51.880	−3.84
BDEs-3-25	30.409	−59.26	45.082	−17.59	51.880	−3.84
BDEs-3-26	30.409	−59.26	45.082	−17.59	51.880	−3.84
BDEs-3-27	30.409	−59.26	45.082	−17.59	51.880	−3.84
BDEs-3-28	30.409	−59.26	45.082	−17.59	51.880	−3.84
BDEs-3-29	30.409	−59.26	45.082	−17.59	51.880	−3.84
BDEs-3-30	84.333	12.98%	54.200	−0.92	64.863	20.23

**Table 4:** Evaluation of flame retardancy and environmental friendliness of PBDEs derivatives.

Compounds	C-Br BDE (kCal/mol)	Relative Error (%)	EC_50_ (mg/L)	Relative Error (%)	lg*BCFs*	Relative Error (%)	VP (Pa)	Relative Error (%)
BDEs-3	95.378	-	0.799	-	5.91 [42]	-	0.109	-
BDEs-3-1	95.965	0.61	11.362 *	-	0.50	−91.54	1.68 × 10^−5^	−99.98%
BDEs-3-2	95.630	0.26	1620.691 *	-	0.50	−91.54	9.30 × 10^−10^	−100.00%
BDEs-3-4	96.191	0.85	0.353	−55.82	3.29	−44.33	1.07 × 10^−2^	−90.18%
BDEs-3-5	96.199	0.86	0.170	−78.72	3.61	−38.92	3.81 × 10^−3^	−96.50%
BDEs-3-6	96.215	0.88	0.092	−88.49	3.89	−34.18	2.74 × 10^−3^	−97.49%
BDEs-3-7	96.264	0.93	0.047	−94.12	4.19	−29.10	1.26 × 10^−3^	−98.84%
BDEs-3-8	96.258	0.92	0.992	24.16	2.32	−60.74	6.34 × 10^−4^	−99.42%
BDEs-3-9	96.090	0.75	2.029	153.94	2.61	−55.84	2.67 × 10^−4^	−99.76%
BDEs-3-10	96.357	1.03	1.609	101.38	2.68	−54.65	1.48 × 10^−3^	−98.64%
BDEs-3-13	96.027	0.68	0.573	−28.29	3.10	−47.55	2.30 × 10^−3^	−97.89%
BDEs-3-19	95.785	0.43	0.417	−47.81	2.82	−52.28	3.52 × 10^−4^	−99.68%

*: The EPI database shows that these compounds may not be soluble and cannot be measured, so the derivative molecules will not be discussed. Ref. [42]: The bioaccumulation data of this molecule comes from this.

**Table 5:** SNR and rank results of 11 factors and 2 levels in the preliminary screening scheme of regulatory factors.

Type Level	Plant Group				Type Level	Microorganism Group			
	1	2	Delta	SNR		1	2	Delta	SNR
A	−13.34	−0.73	12.61	3	A	−31.08	−16.88	14.2	3
B	−8.98	−5.08	3.90	11	B	−32.04	−15.92	16.12	2
C	−11.95	−2.11	9.83	5	C	−28.02	−19.94	8.08	8
D	−11.67	−2.39	9.28	6	D	−33.35	−14.61	18.74	1
E	−10.96	−3.10	7.86	9	E	−28.8	−19.17	9.63	7
F	0.97	−15.03	16.00	2	F	−30.36	−17.61	12.75	5
G	−1.99	−12.07	10.08	4	G	−27.77	−20.2	7.57	4
H	−21.11	7.04	28.15	1	H	−29.6	−18.36	11.24	6
I	−2.99	−11.07	8.08	8	I	−26.73	−21.23	5.49	10
J	−2.73	−11.33	8.60	7	J	−30.47	−17.49	12.98	9
K	−4.27	−9.79	5.52	10	K	−25.07	−22.9	2.17	11

**Table 6:** Molecular dynamics simulation results of regulatory scheme to facilitate the synergistic degradation of PBDEs in soil ^a^.

Sequence	Factor				Plant		Sequence	Microorganism	
	A	D	F	G	Binding Energy (kJ/mol)	Relative Error (%)		Binding Energy (kJ/mol)	Relative Error (%)
0 ^b^	0	0	0	0	−39.964	-	0^b^	−10.186	-
1	0	1	0	1	-	-	1	−26.891	164.00
2	0	0	1	0	-	-	2	-	-
3	0	1	1	0	-	-	3	−5.009	−50.82
4	1	1	0	0	−15.999	−59.97	4	-	-
5	1	0	0	1	−45.742	14.46	5	-	-
6	1	0	1	1	−64.551	61.52	6	−28.054	175.42
7	1	0	1	0	−63.956	60.03	7	-	-
8	1	1	0	1	−25.355	−36.56	8	−14.909	46.37
9	0	1	1	1	−61.141	52.99	9	-	-
10	0	0	0	1	−69.743	74.51	10	−21.361	109.71
11	0	0	1	1	−28.668	−28.27	11	-	-
12	0	1	0	0	−42.800	7.10	12	-	-
13	1	1	1	1	−92.927	132.53	13	−20.589	102.13
14	1	1	1	0	-	-	14	−18.142	78.11
15	1	0	0	0	−81.088	102.90	15	−40.632	298.90

^a^: the smaller the value of binding energy, the greater the binding ability and the better the binding effect; ^b^: sequence 0 is blank control group.

**Table 7:** Docking information statistics of bdes-3 and bdes-3-19 derivatives with plant and microbial degrading enzymes.

Evaluation Project	Plant (1CFN)						Microorganism (1L7V)					
	BDEs-3			BDEs-3-19			BDEs-3			BDEs-3-19		
	Amino Acid Residues	Bond Length	Bond Type	Amino Acid Residues	Bond Length	Bond Type	Amino Acid Residues	Bond Length	Bond Type	Amino Acid Residues	Bond Length	Bond Type
Force	LEU23	5.14 5.14	Alkyl P-Alkyl	PHE91	5.32	P-Alkyl	PRO84	3.77 5.17	P-Alkyl P-Alkyl	PRO84	3.83	P-Alkyl
	VAL27	4.07 4.56	P-Alkyl P-Alkyl	CYS242	3.42 4.16	Br Alkyl	LEU85	4.91	P-Alkyl	LEU85	4.76	Alkyl
	LEU79	4.77	Alkyl	PRO244	4.62	P-Alkyl	PHE86	4.77	P-P	ALA215	3.17	Br
	LYS81	4.94	P-Alkyl	PRO245	4.55	P-Alkyl				LEU147	4.54	Alkyl
	ASP180	3.39	P-Anion	FAD271	3.76 4.13 3.58	P-Alkyl P-Alkyl P-Donor				THR83	3.85	P-Sigma
	LEU182	4.79	P-Alkyl							PRO85	3.27 4.14	Br Alkyl
	LEU183	4.71	Alkyl									
Average Bond length	-	4.61	-	-	4.19	-	-	4.66	-	-	3.93	-
LibDock Score	54.702			68.479			53.951			68.345		

## Supplementary Materials

The following are available online, **Table S1** Structures of PBDEs that are mentioned in the introduction, **Table S2** References on comprehensive evaluation of pollutant properties using mathematical methods, **Table S3** Horizontal comparison of model parameters, **Table S4** Molecular information of PBDEs derivatives, **Table S5-1,2,3** Toxicokinetic prediction and assessment of BDE before and after modification using TOPKAT module.
