# Supplementary material for: Molecular Modifications and Control of Processes to Facilitate the Synergistic Degradation of Polybrominated Diphenyl Ethers in Soil by Plants and Microorganisms Based on Queuing Scoring Method
Source: Molecules. 2021 Jun 26;26(13):3911. doi: 10.3390/molecules26133911 (PMC8271410; doi:10.3390/molecules26133911)
Supplement: Supplementary file 1 [file molecules-26-03911-s001.zip › molecules-1222659-supplementary.pdf]

Table S1 Structures of PBDEs that are mentioned in the introduction

References	Molecules	Structure
[9]	BDE-28	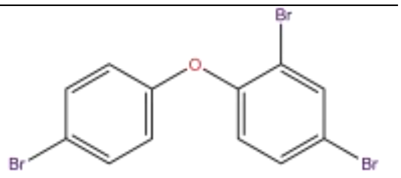
	BDE-47	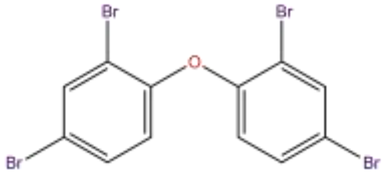
[10]	BDE-209	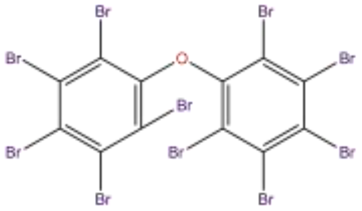
[12]	BDE-3	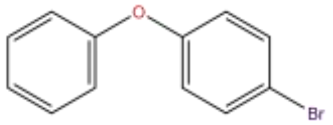
	BDE-8	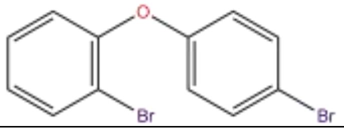

Table S2 References on comprehensive evaluation of pollutant properties using mathematical methods

References	Method
[S1]	Averaging method
[S2]	Threshold Method
[S3]	Variation Weighting Method
[S4]	Simplified formula method
[S5]	Fuzzy comprehensive evaluation

Table S3 Horizontal comparison of model parameters

References	Model	q^2 (>0.5)	$r^2 pred$ (>0.6)
[S1]	CoMSIA	0.535	0.773
[S2]	CoMFA	0.760	0.650
[S3]	CoMFA	0.767	0.967
[S4]	CoMFA	0.691	0.833
	CoMSIA	0.828	0.838
[S5]	CoMFA	0.560	0.692

Table S4 Molecular information of PBDEs derivatives

No.	Structural formula	Structure	Molar mass (g/mol)
BDE-3	$C_{12}H_9BrO$	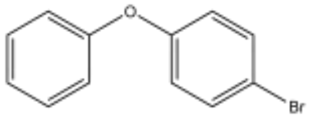	249.10
BDE-3-1	$C_{13}H_9BrO_3$	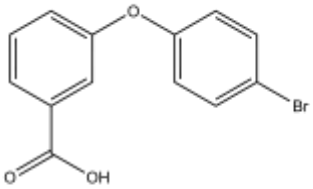	293.11
BDE-3-2	$C_{12}H_9BrO_4S$	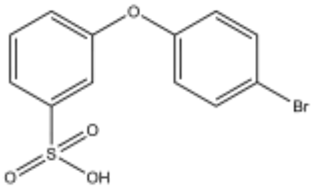	329.17
BDE-3-3	$C_{12}H_{10}BrO_5P$	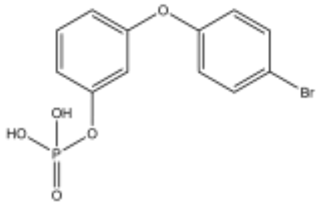	345.08
BDE-3-4	$C_{13}H_{11}BrO$	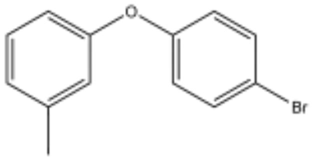	263.13
BDE-3-5	$C_{14}H_{13}BrO$	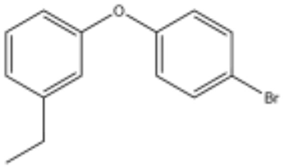	277.16
BDE-3-6	$C_{15}H_{15}BrO$	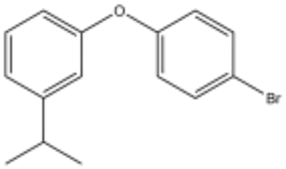	291.18
BDE-3-7	$C_{16}H_{17}BrO$	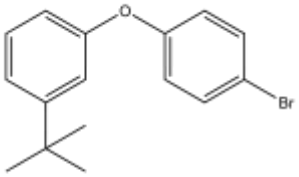	305.21
BDE-3-8	$C_{12}H_{10}BrNO$	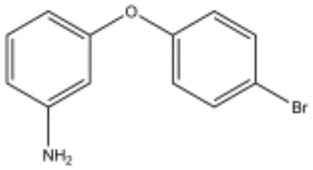	264.12

BDE-3-9	$C_{12}H_9BrO_2$	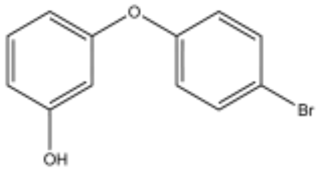	265.10
BDE-3-10	$C_{13}H_{12}BrNO$	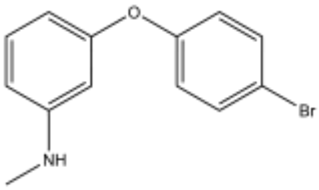	278.14
BDE-3-11	$C_{13}H_{11}BrO_2$	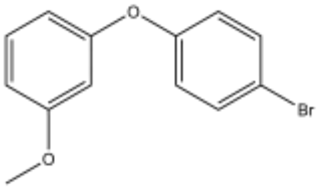	279.13
BDE-3-12	$C_{13}H_8BrNO$	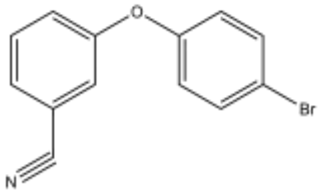	274.11
BDE-3-13	$C_{14}H_9BrO$	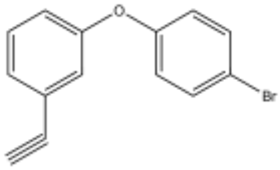	273.12
BDE-3-14	$C_{14}H_{11}BrO$	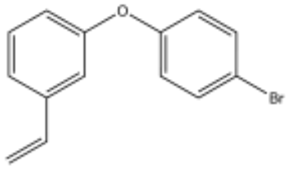	275.14
BDE-3-15	$C_{14}H_9BrO$	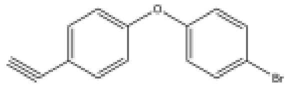	273.12
BDE-3-16	$C_{12}H_9BrO2$	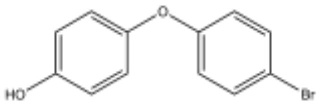	265.10
BDE-3-17	$C_{13}H_{11}BrO_2$	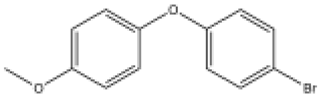	279.13
BDE-3-18	$C_{14}H_{11}BrO$	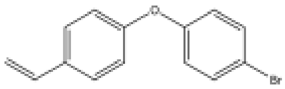	275.14
BDE-3-19	$C_{14}H_{11}BrO_3$	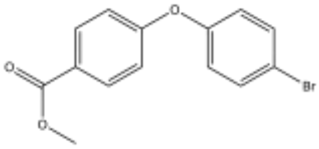	307.14

BDE-3-20	$C_{14}H_{11}BrO_3$	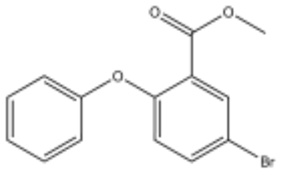	307.14
BDE-3-21	$C_{14}H_{11}BrO_3$	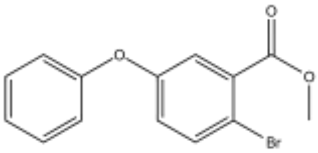	307.14
BDE-3-22	$C_{14}H_9BrO$	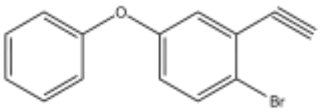	273.12
BDE-3-23	$C_{13}H_{11}BrO_2$	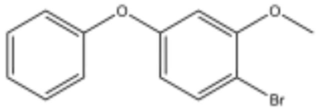	279.13
BDE-3-24	$C_{12}H_9BrO_2$	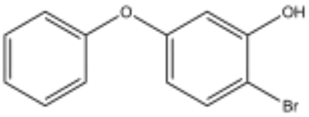	265.10
BDE-3-25	$C_{14}H_{11}BrO$	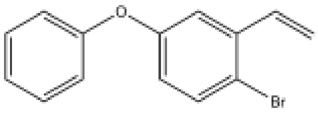	275.14
BDE-3-26	$C_{14}H_{11}BrO$	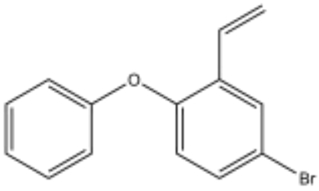	275.14
BDE-3-27	$C_{13}H_{11}BrO_2$	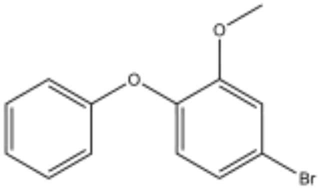	279.13
BDE-3-28	$C_{12}H_9BrO_2$	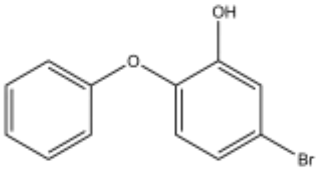	265.10
BDE-3-29	$C_{14}H_9BrO$	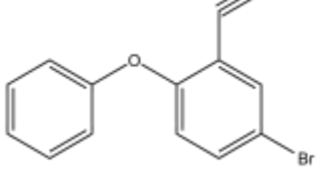	273.12
BDE-3-30	$C_{12}H_{11}BrOSi$	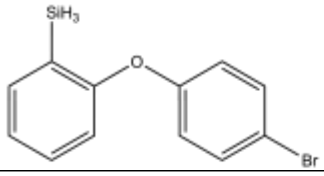	279.20

Table S5-1 Toxicokinetic prediction and assessment of BDE before and after modification using TOPKAT module

**No.**	**Probability value**								
	Ames mutagenicity	NTP Rodent Carcinogenicity				FDA Rodent Carcinogenicity			
		Male rat	Female rat	Male mouse	Female mouse	Male rat Non vs. Carc	Male rat Single vs. Mult	Female rat Non vs. Carc	Female rat Single vs. Mult
BDE-3	0.6454/Non	0.6695/Carc	0.5620/Carc	0.8748/Carc	0.7095/Carc	0.3204/Non	Needless	0.2630/Non	Needless
BDE-3-4	0.6996/Non	0.6127/Carc	0.5333/Carc	0.7729/Carc	0.5946/Non	0.2811/Non	Needless	0.2361/Non	Needless
BDE-3-5	0.6472/Non	0.5567/Non	0.5142/Non	0.8151/Carc	0.7558/Carc	0.3223/Non	Needless	0.2471/Non	Needless
BDE-3-7	0.6455/Non	0.6587/Carc	0.5277/Non	0.5574/Non	0.3541/Non	0.3042/Non	Needless	0.2196/Non	Needless
BDE-3-13	0.4206/Non	0.5420/Non	0.5059/Carc	0.7859/Carc	0.6016/Carc	0.3047/Non	Needless	0.2385/Non	Needless
BDE-3-19	0.6418/Non	0.4707/Non	0.5010/Non	0.8600/Carc	0.5851/Non	0.2935/Non	Needless	0.2492/Non	Needless

Table S5-2 Toxicokinetic predictions of BDE before and after modification using TOPKAT module

No.	**Probability value**				Rat chronic oral LOAEL* g/kg	Rat oral LD50* g/kg	Rat maximum tolerated dose-feed/Water g/kg	Rat maximum tolerated dose-gavage g/kg	Rat inhalational LC50 * $mg/m^3/h$
	FDA Rodent Carcinogenicity								
	Male mouse Non vs. Carc	Male mouse Single vs. Mult	Female mouse Non vs. Carc	Female mouse Single vs. Mult					
BDE-3	0.3056/Carc	0.1464/Single	0.2758/Carc	0.4973/Mult	0.1490/Mod	0.7420/Low	0.0727/Mod	0.0006/Hyper	3310.7200/High
BDE-3-4	0.2822/Carc	0.1457/Single	0.2795/Carc	0.4941/Mult	0.1342/Mod	0.4853/Low	0.2464/Mod	0.0002/Hyper	3475.5200/High
BDE-3-5	0.2884/Carc	0.1458/Single	0.3146/Carc	0.4444/Mult	0.0426/High	2.2460/Non	0.0946/Mod	0.0010/Hyper	5211.2700/High
BDE-3-7	0.2118/Non	Needless	0.2101/Non	Needless	0.0620/Mod	4.5981/Non	0.1444/High	0.0004/Hyper	8941.2300/Mod
BDE-3-13	0.2615/Carc	0.1500/Single	0.2159/Non	Needless	0.0174/High	0.7351/Low	0.1437/Mod	0.0001/Hyper	466.9520/Hyper
BDE-3-19	0.2841/Carc	0.1456/Single	0.2547/Carc	0.5429/Mult	0.0969/Mod	0.9813/Low	0.2464/Mod	0.0002/Hyper	3475.5200/High

* Assessment finished based on “Acute Oral, Inhalation and Percutaneous Toxicity Classification for Rats” (Table S4-2), cited form the Guidelines for the Hazard Evaluation of New Chemical Substances (HJ/T154-2004), Ministry of Ecology and Environment of the People's Republic of China, April 13, 2004.

Table S5-3 Toxicokinetic predictions of BDE before and after modification using TOPKAT module

No.	Fathead minnow LC50 * g/L	Daphnia EC50 * mg/L	Probability value					
			Developmental toxicity potential (DTP)	Skin irritancy	Skin sensitization NON vs. SENS	Skin sensitization (MLD/MOD) vs. SEV	Ocular irritancy (Non/Mild) vs. (Mod/Sev)	Ocular irritancy MOD vs. SEV
BDE-3	0.0019/Hyper	0.1466/Hyper	0.6253/Yes	0.9635/Non	0.8855/SENS	0.9866/SEV	0.7970/Mild	Needless
BDE-3-4	0.0014/High	0.1218/Hyper	0.6590/Yes	0.9633/Non	0.8433/SENS	0.9894/SEV	0.8146/(Mod/Sev)	0.6743/Mod
BDE-3-5	0.0022/Hyper	0.2380/Hyper	0.6551/Yes	0.9568/Non	0.8792/SENS	0.9837/SEV	0.7238/Mild	Needless
BDE-3-7	0.0020/Hyper	0.2376/Hyper	0.6549/Yes	0.9688/Non	0.8767/SENS	0.9917/SEV	0.7758/Mild	Needless
BDE-3-13	0.0058/High	0.1118/Hyper	0.5659/Yes	0.9506/Non	0.8797/SENS	0.9875/SEV	0.8372/(Mod/Sev)	0.6974/Sev
BDE-3-19	0.0034/High	0.1510/Hyper	0.6587/Yes	0.9554/Non	0.8501/SENS	0.9926/SEV	0.8491/(Mod/Sev)	0.6816/Sev

* Assessment finished based on "Ecotoxicological Hazard Classification" (Table S4-3), cited form the Guidelines for the Hazard Evaluation of New Chemical Substances (HJ/T154-2004), Ministry of Ecology and Environment of the People's Republic of China, April 13, 2004.

References:

[S1] Hou, Y.L., Zhao, Y.Y., Li, Q., Li, Y. Highly biodegradable fluoroquinolone derivatives designed using the 3D-QSAR model and biodegradation pathways analysis[J]. Ecotox. Environ. Safe., 2020, 191, 110186.

[S2] Yang, L.Z., Li, M.H., Liu, M. Establishment of a CoMFA Model Based on the Combined Activity of Bioconcentration, Long-Range Transport, and Highest Infrared Signal Intensity and Molecular Design of Environmentally Friendly PBB Derivatives[J]. Polymers, 2021, 13, 356.

[S3] Sun, P.X.; Zhao, Y.Y.; Yang, L.Z.; Ren, Z.X.; Zhao, W.J. Environmentally Friendly Quinolones Design for a Two-Way Choice between Biotoxicity and Genotoxicity through Double-Activity 3D-QSAR Model Coupled with the Variation Weighting Method[J]. Int. J. Env. Res. Pub. He., 2020, 17(24), 1-22.

[S4] Li, Q., Qiu, Y.L., Li, Y. Molecular Design of Environment-Friendly PAE Derivatives Based on 3D-QSAR Assisted with a Comprehensive Evaluation Method Combining Toxicity and Estrogen Activities[J]. Water Air Soil Poll., 2020, 231, 194.

[S5] Ren, Z.X., Wang, Y.W., Xu, H.H., Li, Y.F., Han, S. Fuzzy Comprehensive Evaluation Assistant 3D-QSAR of Environmentally Friendly FQs to Reduce ADRs[J]. Int. J. Env. Res. Pub. He., 2019, 16(17): 3161.

[S6] Xia, X., Maliski, E., Gallant, P., et al. Classification of kinase inhibitors using a Bayesian model [J]. Journal of Medicinal Chemistry, 2004, 47(18): 4463-4470.

[S7] Burton, J., Iijaali, I., Petite, F., et al. Virtual Screening for Cytochromes P450: Successes of Machine Leaming Filters [J]. Combinatorial Chemistry & High Throughput Screening, 2009, 12(4): 369-382.

[S8] Obach, R. Aldehyde oxidase in drug design and drug discovery [J]. Drug Metabolism and Pharmacokinetics, 2018, 33(1). DOI: 10.1016/j.dmpk.2017.11.053.

[S9] Fu, X., Wang, G., Shan, H., et al. Predicting blood-brain barrier penetration from molecular weight and number of polar atoms [J]. European Journal of Pharmaceutics & Biopharmaceutics, 2008, 70(2): 462-466.
